# Pituitary Hyperplasia Due to Longstanding Primary Hypothyroidism: A Case Report and Comprehensive Review of the Literature

**DOI:** 10.3390/biomedicines12061368

**Published:** 2024-06-19

**Authors:** Anna Roux, Daniela Rosso, Daniela Cuboni, Mauro Maccario, Silvia Grottoli, Emanuela Arvat, Valentina Gasco

**Affiliations:** 1 Division of Oncological Endocrinology, Department of Medical Sciences, University of Turin, 10126 Turin, Italy; anna.roux@edu.unito.it (A.R.); rosso.daniela91@gmail.com (D.R.); emanuela.arvat@unito.it (E.A.); 2 Division of Endocrinology, Diabetes and Metabolism, Department of Medical Sciences, University of Turin, 10126 Turin, Italy; daniela.cuboni@unito.it (D.C.); mauro.maccario@unito.it (M.M.); silvia.grottoli@unito.it (S.G.)

**Keywords:** primary hypothyroidism, pituitary hyperplasia, pituitary adenoma, levothyroxine replacement therapy, nipple sign

## Abstract

Hypothyroidism is a frequently diagnosed endocrine disorder. Common signs and symptoms include fatigue, cold intolerance, hoarseness, dry skin, constipation, a slow relaxation phase of deep tendon reflexes, and bradycardia. However, some patients may exhibit atypical signs and symptoms, which can result in diagnostic confusion. Pituitary hyperplasia resulting from longstanding primary hypothyroidism was first described by Niepce in 1851. It is usually asymptomatic, but sometimes, in addition to symptoms of overt hypothyroidism, patients may complain of headaches, hypopituitarism, visual field impairment, and hyperprolactinemia. Furthermore, on imaging, pituitary hyperplasia can be mistaken for a pituitary adenoma. Distinguishing between the two is crucial, as their management differs; the former often responds to thyroid hormone replacement therapy, while the latter might need treatment with surgery and/or radiotherapy. Here we describe a patient who developed pituitary hyperplasia in the setting of longstanding uncompensated primary hypothyroidism due to a lack of compliance with levothyroxine replacement therapy. We also review the clinical, laboratory, and radiologic findings of the case reports available in the literature up to now in order to improve the knowledge and the care of the disease.

## 1. Introduction

Primary hypothyroidism (PH) is a frequently diagnosed endocrine disorder [1]. Common signs and symptoms include asthenia, lethargy, cold sensitivity, rough voice, dry skin, constipation, a slow relaxation phase of deep tendon reflexes, and decreased heart rate [1]. However, some subjects may present atypical signs and symptoms, which can result in diagnostic confusion.

Pituitary hyperplasia is an under-recognized manifestation that can occur quite frequently in PH. It was first described by Niepce in 1851 [2] and, subsequently, additional case reports have been reported in the literature and reviewed by Beck-Peccoz in a comprehensive review in 1996 [3].

In PH, low circulating thyroid hormone levels result in insufficient negative feedback on the hypothalamus, leading to increased thyrotropin-releasing hormone (TRH). High TRH levels induce thyrotroph cell hyperplasia in the pituitary gland and can also prompt lactotroph cell hyperplasia, thereby causing elevated prolactin (PRL) in affected individuals [4]. Moreover, transdifferentiation of somatotrophs and lactotrophs into cells secreting thyroid stimulating hormone (TSH) may contribute to pituitary hyperplasia [5,6].

Pituitary imaging is not typically performed in adult PH, so the frequency and extent of pituitary hyperplasia due to thyroid dysfunction is not known. Certain studies have shown that the incidence of pituitary hyperplasia in PH can vary anywhere from 25% to 81% [3]. Furthermore, a correlation has been reported between the presence of pituitary hyperplasia, as measured by the size of the sella turcica, and TSH levels [7].

The presentation and imaging may resemble that of a pituitary tumor: patients may exhibit symptoms of hypothyroidism [8], as well as hyperprolactinemia, headaches, and, in some cases, visual field defects if the enlarged pituitary gland compresses the optic chiasm. Some magnetic resonance imaging (MRI) signs can differentiate between hyperplasia and pituitary adenoma, and the most indicative one is the projection of the median line of the pituitary mass with a smooth edge, referred to as the ‘nipple sign’ or ‘dome-shaped pituitary enlargement’ [9].

The diagnosis of pituitary hyperplasia induced by PH is crucial, as its treatment is thyroid hormone replacement and not surgery [3,10].

Here we describe a patient who developed pituitary hyperplasia in the setting of longstanding uncompensated PH due to a lack of compliance with levothyroxine replacement therapy. 

To gather further information about this clinical feature, we conducted a review of case reports available in the literature since 1996, examining clinical, laboratory, and radiological findings. We selected this starting date because in 1996, a comprehensive review of pituitary hyperplasia cases related to uncompensated PH was published [3]. In that review, Beck-Peccoz et al. analyzed 210 published cases (48 children and 152 adults) from 97 studies. The authors noted significant variations in methodology and focus across the studies, likely influenced by the publication year. In fact, the introduction of computerized axial tomography (CT) and, later, MRI as imaging techniques for the hypothalamic-pituitary area occurred gradually during the 1970s and 1980s, thus replacing sella turcica radiography due to their ability to provide more detailed images and better spatial resolution for a more accurate assessment of the anatomy and pathologies involving the hypothalamus and pituitary.

Moreover, it must be considered that the third-generation TSH assay was introduced during the 1990s. This technology allowed for greater accuracy in measuring TSH levels, thereby improving the diagnosis and treatment of thyroid disorders.

## 2. Materials and Methods

### 2.1. Literature Search

We searched Pubmed and EMBASE to conduct a comprehensive review of literature for cases of pituitary hyperplasia in PH adult patients. The search terms used included ‘primary hypothyroidism’ and ‘pituitary hyperplasia’ or ‘pituitary adenoma’. No limitations were made to make the search as sensitive as possible. The filter for studies involving adult subjects belonging to the human species was used. The reference lists of identified studies and key review articles, including previously published reviews, were also examined. Only articles in English were included. Searches were conducted from January 1996 up to December 2023.

### 2.2. Study Selection

For inclusion, studies were required to fulfill the following: (i) focus on cases of overt PH; (ii) report at least the TSH levels at diagnosis; and (iii) report at least the characteristics of the pituitary lesion at diagnosis (either in terms of diameter(s) or volume, or at least in terms of radiological description).

We excluded the following from the analysis: (i) cases with radiological investigation other than CT or MRI; (ii) pediatric cases; (iii) studies where hormonal values and pituitary size dimensions were reported as the mean of multiple observations and not for individual subjects; and (iv) conference abstracts.

### 2.3. Identification of Relevant Studies

Potentially relevant papers were identified through the examination of titles and abstracts. If abstracts were unavailable or insufficient in providing results, the entire article was retrieved and screened to assess its eligibility for inclusion.

### 2.4. Data Extraction and Synthesis

A registration form was created to assess if individual studies fulfilled the eligibility criteria and to gather data. Two investigators independently evaluated and extracted data from the papers based on the predefined criteria. Any discrepancies were resolved through consensus. The extracted data included author and date of publication, epidemiological data (gender and age at diagnosis), clinical presentation (symptoms related to PH, symptoms related to mass effect, systemic symptoms), TSH and PRL levels, pituitary function in terms of partial or total hypopituitarism, type of radiological investigation (CT and/or MRI), treatment, and outcome (type of therapy and outcome at follow-up, when available). 

Hormonal status details were derived from each study, and hypopituitarism was defined according to the criteria outlined in each respective paper. The diagnosis of hyperprolactinemia was established based on prolactin levels exceeding the upper limit of the normal reference range for gender; moreover, in consideration of the extreme variability of normal limits among different hormonal assays used in various studies, for the purpose of comparisons and statistical analyses, TSH and PRL levels were expressed as ‘upper limit normal’ (ULN).

For radiological findings, nipple sign or dome-shape, intra-, supra-, and/or infra-sellar extension of the pituitary lesion, invasion of the cavernous sinus, compression of the optic chiasm, or other radiological features reported in the case description were recorded. The maximum diameter or volume of the pituitary mass was considered; if the volume was not provided in the study but the three diameters were available, the volume of the lesion was calculated according to the simplified ellipsoid equation formula (0.5 × width × length × height) [11].

We also described a new case of pituitary hyperplasia due to longstanding PH diagnosed and treated in our hospital (AOU Città della Salute e della Scienza di Torino, Italy).

### 2.5. Statistical Analysis

Normality was assessed utilizing the Shapiro–Wilk test. Variables that were not normally distributed and categorical data were presented as median and interquartile range (IQR) or counts and percentages, respectively. The Wilcoxon test was employed to compare paired variables and the Kruskal-Wallis test for unpaired variables. Spearman’s test was utilized to assess the correlation of continuous variables.

Statistical analysis was performed using MedCalcTM^®^ (Statistical Software version 20.007, MedCalc Software Ltd., Ostend, Belgium).

## 3. Results

**Figure 1:**
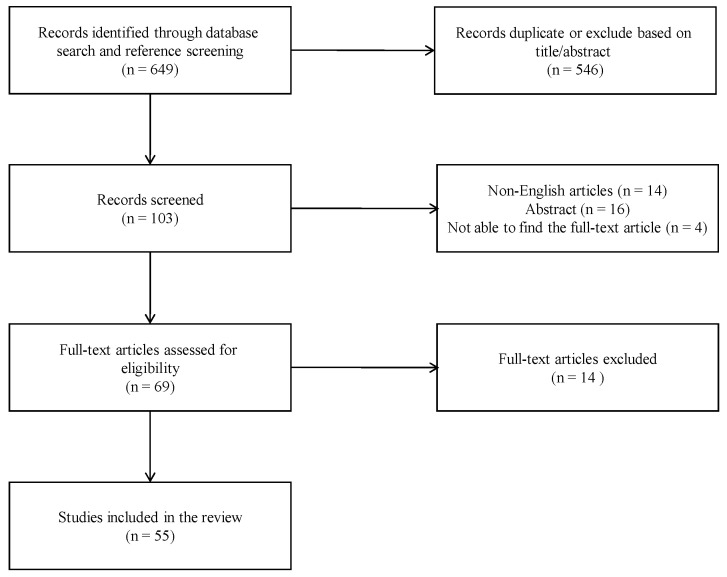
Flowchart of studies’ inclusion.

The literature search yielded 649 potentially relevant articles, spanning from January 1996 to December 2023. Following the removal of duplicates and the review of titles, abstracts, and full-length articles, we identified 55 articles [6,9,12,13,14,15,16,17,18,19,20,21,22,23,24,25,26,27,28,29,30,31,32,33,34,35,36,37,38,39,40,41,42,43,44,45,46,47,48,49,50,51,52,53,54,55,56,57,58,59,60,61,62,63,64] for further examination, all of which were included in our analysis (Figure 1). The combined data encompassed a total of 92 subjects (for details, also see Supplementary Materials, Table S1). Additionally, we provide details of a newly diagnosed and treated case from our hospital.

### 3.1. Case Description

**Figure 2:**
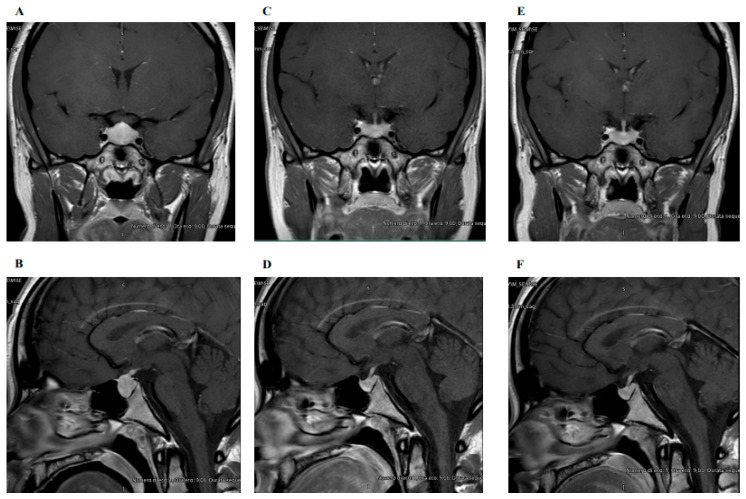
Coronal (**A**) and sagittal (**B**) post-contrast T1-weighted MRI pituitary scan at diagnosis, showing pituitary enlargement with dome-shaped convexity, close to the optic chiasm. Coronal (**C**) and sagittal (**D**) post-contrast T1-weighted MRI pituitary scan 5 months after the first normalization of thyroid function, indicating a reduction in the size of the entire pituitary gland. Coronal (**E**) and sagittal (**F**) post-contrast T1-weighted MRI pituitary scan 12 months after the first normalization of thyroid function: the pituitary volume remained almost unchanged compared to the previous evaluation.

A 22-year-old female was referred to our attention for the evaluation of a pituitary lesion not well-characterized on a brain MRI, which was performed for an episode of lipothymia associated with headache and oligomenorrhea. To better evaluate the lesion, a pituitary MRI was performed (Figure 2), which revealed an oval-shaped formation of 20 mm in size, located intra, infra, and suprasellar, characterized by an isointense signal on T1, an iso-hypointense signal on T2, and a homogeneous gadolinium contrast enhancement (CE), suggestive for pituitary macroadenoma. The pituitary stalk was horizontalized, and the lesion, approaching the optic chiasm, did not seem to determine its compression. No visual field alteration was observed.

The patient had undergone a total thyroidectomy 8 years before for Basedow’s disease and claimed to assume oral levothyroxine in the morning on an empty stomach.

Laboratory tests showed a severe PH, with a remarkable increase in TSH (>300 µIU/mL, normal values 0.35–4.94 µIU/mL) and a decrease in thyroid hormones (fT3 1.4 ng/L, normal values 1.6–3.9 ng/L; fT4 4.5 ng/L, normal values 7–14.8 ng/L), despite the generous dose of daily levothyroxine that the patient reported to take (>1.9 µg/kg/day). Furthermore, a secondary hypogonadism associated with PRL levels at the upper normal range (PRL 25.7 µg/L, normal values 3.5–26 µg/L) and an elevated alpha subunit (1.6 mIU/mL, normal values 0.1–1 mIU/mL) were found, while both basal and stimulated adrenal axis function and IGF-I levels were normal. 

On physical examination, she had a body mass index of 26.4 kg/m^2^, blood pressure of 110/70 mmHg, and a regular heart rate (70 beats per minute). She complained of asthenia, headache, tingling in the extremities, and secondary oligomenorrhea. No other signs of hypothyroidism or other pituitary dysfunction were documented. 

The case was discussed in the multidisciplinary Pituitary Unit: since pituitary hyperplasia secondary to uncontrolled hypothyroidism could not be excluded, before proceeding with a neurosurgical evaluation, which was initially taken into account for the presumed diagnosis of pituitary macroadenoma, a repetition of pituitary MRI was recommended after the normalization of TSH levels.

After excluding the presence of laboratory interferences (macro-TSH and heterophilic antibodies) and of the main causes of bowel malabsorption (negative anti-transglutaminase Ab and anti-parietal cell Ab) and after documenting correct levothyroxine absorption kinetics, the patient was proposed to take 1000 μg of levothyroxine in a weekly oral single dose under medical supervision for 6 weeks, with evaluation of TSH and thyroid hormone levels before each administration (Table 1). 

After 3 weeks of therapy, laboratory tests showed a normalization of thyroid function. Furthermore, normal menstrual cycles reappeared.

Pituitary MRIs at 5 and 12 months after TSH normalization showed significant volumetric reduction of the pituitary gland (Figure 2). The signal intensity in the pituitary was uniform, and no abnormalities of the pituitary stalk or the optic chiasm were described. 

The shrinkage of the pituitary lesion consequently to TSH reduction confirmed the hypothesis of pituitary hyperplasia secondary to uncontrolled PH, thus avoiding the need for neurosurgical intervention. 

The multiple attempts to shift the levothyroxine therapy to an equivalent daily oral dose resulted in a new onset of hypothyroidism, with restoration of euthyroidism only after switching again to the weekly single dose. Nevertheless, the patient never admitted to being non-compliant.

### 3.2. Comprehensive Review

Since 1996, 93 clinical cases (20 M; age, median 32 (IQR 24.5) years, range 18–83 years) of pituitary hyperplasia due to longstanding PH in adult subjects have been described in the English literature, including our patient.

#### 3.2.1. Clinical and Hormonal Features

The main complaints at presentation, prompting the patient to seek medical attention, are reported in Table 2. 

The time of evaluation since the first symptom (*n* = 40) was 8.5 (20) months (range 0–132 months). The pathogenesis of hypothyroidism was specified in 51.6% of cases (*n* = 48): autoimmune hypothyroidism was the most frequent cause (54.2%), while a post-surgical or post-radiometabolic therapy cause was reported in 35.4% and 4.2%, respectively. The diagnosis of hypothyroidism dated back (*n* = 26) 0.75 (107.3) months (range 0.75–408 months). A goiter, diagnosed by clinical or ultrasound examination, was present in 21.9% of patients (*n* = 32).

Median TSH level (*n* = 67) at diagnosis was 33.5 (104) × ULN (range 1.5–836.6 × ULN), while median PRL level (*n* = 73) was 1.6 (2.3) × ULN (range 0.1–204.8 × ULN); hyperprolactinemia, usually moderate, was present in 69.1% (*n* = 56/81) of patients.

Although there was no significant correlation between TSH and PRL levels (*n* = 58), TSH was significantly (*p* = 0.031) higher in hyperprolactinemic patients (*n* = 40; 48.9 (123.6) × ULN) compared to normo-prolactinemic patients (*n* = 18; 23.9 (24.9) × ULN).

At diagnosis, alpha-subunit levels were reported, and a TRH test was performed in five patients only, and thus, any further analysis of the data appeared unnecessary.

Some degree of hypopituitarism was reported in 23.9% (*n* = 11/46) of cases.

Some alteration of the visual field was present in 37.5% of patients undergoing specific evaluation (*n* = 32).

#### 3.2.2. Imaging Features

An MRI was performed in 92 patients, while a CT study was performed in 10 patients; 9 out of 93 patients underwent both MRI and CT scans. 

In 58.6% (*n* = 34/58) of cases, pituitary hyperplasia was initially interpreted as a pituitary adenoma. Table 3 summarizes the results of pituitary MRI/CT at baseline: a mass with suprasellar extension was the most frequent finding (96.5%; *n* = 56/58), and a compression of the optic chiasm was highlighted in 70.4% (*n* = 38/54); an invasion of the cavernous sinus was reported in 17.2% (*n* = 10/58), while an infrasellar extension of the mass was reported in 10.3% (*n* = 6/58). A nipple sign or a dome-shaped pituitary was present in 66.7% of the evaluable cases (*n* = 24/36).

The median pituitary maximal mass diameter (*n* = 40) was 16.5 (7) mm (range 8.0–71 mm); the median pituitary mass volume (*n* = 59) was 900 (979) mm^3^ (range 300–87969 mm^3^).

There was not a significant correlation between pituitary maximal mass diameter and TSH × ULN (*n* = 25; *r* = 0.10, *p* = 0.63) or PRL × ULN (*n* = 30; *r* = −0.05, *p* = 0.79) as well as between pituitary mass volume and TSH × ULN (*n* = 41; *r* = 0.12, *p* = 0.44) or PRL × ULN (*n* = 49; *r* = 0.0003, *p* = 0.99).

Nevertheless, pituitary mass volume was significantly higher in patients with TSH levels > 100 μIU/mL (*n* = 40; 1040 (949) mm^3^) compared to that in patients with TSH levels ≤ 100 μIU/mL (*n* = 19; 539 (597.7) mm^3^) (*p* = 0.015) as well as in hyperprolactinemic patients (*n* = 32; 1082 (1278) mm^3^) compared to normo-prolactinemic patients (*n* = 20; 542.5 (252) mm^3^) (*p* = 0.0006).

Furthermore, a direct, significant correlation was present between the time elapsed since the diagnosis of hypothyroidism and both pituitary maximal mass diameter (*n* = 9; *r* = 0.93, *p* = 0.0003) and pituitary mass volume (*n* = 20; *r* = 0.8, *p* < 0.0001).

#### 3.2.3. Treatment

Table 4 summarizes the treatments undergone by the 93 analyzed patients. The majority were treated by initiating or adjusting levothyroxine therapy. However, 11 patients were referred for neurosurgical intervention, i.e., in 8 cases, surgery was performed before any thyroid hormone replacement [6,9,13,22,39,44,63], while in 2 cases surgery became necessary due to worsening conditions or failure to achieve full normalization during levothyroxine treatment [25,51]. In one case, surgery was performed simultaneously with the initiation of levothyroxine therapy to prevent further visual field alterations [43]. Histological examination revealed pituitary hyperplasia in five cases [6,9,13,44,63], while it allowed for the identification of pure (two cases) or multi-hormonal TSH-secreting adenoma (four cases) in the remaining six cases [22,25,39,43,51].

#### 3.2.4. Outcome

A hormonal follow-up was available in 60% of the patients. The time elapsed for hormonal re-evaluation at follow-up (*n* = 41) was 4.0 (4.0) months (range 0.25–72 months). The median levels of TSH at follow-up (*n* = 48) were 0.58 (0.65) × ULN (range 0.002–38.2 × ULN), while the median levels of PRL (*n* = 36) were 0.45 (0.67) × ULN (range 0.11–5.2 × ULN); both were significantly reduced compared to values at diagnosis (*p* < 0.0001 for both).

At follow-up, TSH levels (*n* = 49) were normalized in 35 patients, reduced in 5, and still elevated in 9 patients. PRL levels (*n* = 37) were normal in 28 patients and still elevated in 9 patients.

MRI follow-up was performed in 86% of the patients. The time elapsed for MRI re-evaluation at follow-up (*n* = 41) was 4.0 (5.0) months (range 1–48 months). Median pituitary maximal mass diameter at follow-up (*n* = 16) was 8.8 (3.8) mm (range 5.5–42 mm), while median pituitary mass volume at follow-up (*n* = 37) was 385 (272.3) mm^3^ (range 94.5–25,830 mm^3^); both were significantly reduced compared to values at diagnosis (*p* < 0.0001 for both).

At follow-up, pituitary MRI re-evaluation (*n* = 77) showed complete regression of the lesion in 55 cases and partial resolution in 17 cases, respectively. Stability or further increase of the pituitary lesion was reported in four and one patients, respectively. 

In the only case of further pituitary volume increase [34] and in two out of four cases of morphological stability [31,34,46], lack of compliance with levothyroxine therapy was reported [31,34]. In a case of ongoing morphological stability during therapy with levothyroxine, suspicion of prolactinoma led to the initiation of bromocriptine therapy, with evidence of a subsequent response confirming the diagnostic hypothesis [46].

Despite the marked reduction of the pituitary lesion during levothyroxine therapy, doubt arose regarding the coexistence of a TSH-secreting macroadenoma, later confirmed on histological examination, due to the persistent, significant volumetric increase of the pituitary in one case [25]. In another case, the suspicion of a non-functioning macroadenoma arose, though whether surgical intervention was subsequently performed is not known [40].

At follow-up, no significant correlation was found between the pituitary maximal mass diameter and TSH × ULN (*n* = 12; *r* = −0.24, *p* = 0.46) or PRL × ULN (*n* = 7; *r* = 0.16, *p* = 0.74). However, a significant direct correlation was observed between pituitary mass volume and PRL × ULN (*n* = 16; *r* = 0.6, *p* = 0.014), but not between pituitary mass volume and TSH × ULN (*n* = 18; *r* = 0.01, *p* = 0.96). 

Furthermore, there was a significant direct correlation between the time elapsed for MRI re-evaluation and both pituitary maximal mass diameter (*n* = 15; *r* = 0.82, *p* = 0.0002) and pituitary mass volume (*n* = 4; *r* = 0.96, *p* = 0.038) at follow-up.

## 4. Discussion

In this literature review, we have updated the cases of pituitary hyperplasia in adult subjects with chronically uncompensated PH, adding 93 new cases to the previous 152 cases reported by Beck-Peccoz in the 1996 review [3]. Our work, analyzing the most recent literature:

(i) Reports the clinical, hormonal, and radiological characteristics at the diagnosis of pituitary hyperplasia in the context of long-standing overt PH.
(ii) Emphasizes the good response to therapy with levothyroxine, reporting a complete resolution of the condition in about 70% of the cases.
(iii) Underscores that it is still possible, in a significant percentage of cases, to make the mistake of referring such patients to neurosurgery with possible irreversible damage, especially considering the young age of these subjects.
(iv) Highlights that, even if rare, a pituitary adenoma should not be ruled out entirely: in case of evidence of unresponsiveness to levothyroxine therapy, the presence of a non-functioning pituitary adenoma, a PRL-secreting pituitary adenoma, and even a TSH-secreting pituitary adenoma must be considered.

Hypothyroidism manifests with a wide range of subtle and nonspecific symptoms such as weight gain, dry skin, muscle weakness, constipation, cold intolerance, brittle hair, poor concentration, depression, fatigue, menstrual irregularities, and more [1]. However, there are also uncommon symptoms documented in medical literature. Since their exact prevalence within hypothyroid populations has not been epidemiologically studied, they frequently pose diagnostic challenges. Patients exhibiting these symptoms are often misdiagnosed due to a lack of awareness among primary care providers, resulting in significant delays in treatment and suboptimal therapeutic results.

Pituitary hyperplasia is an under-recognized manifestation that can occur quite frequently in PH. In our literature review of the most recent cases of pituitary hyperplasia due to long-standing hypothyroidism, the presenting signs and symptoms showed significant variability, including common symptoms of hypothyroidism (such as anemia, constipation, fatigue, weight gain, cold intolerance, and bradycardia), as well as irregular menses and galactorrhea suggestive of hyperprolactinemia, or compressive symptoms such as headache and visual disturbances. However, very rare symptoms such as ovarian hyperstimulation syndrome [16,32,33,59,62,64] or psychosis [28,38], as well as pseudoacromegaly [20,37], were also reported.

Although hypothyroidism is a rare cause of ovarian hyperstimulation syndrome, this condition has been reported as the main symptomatology leading to the diagnosis of pituitary hyperplasia in 13% of the female patients reviewed by us. Different theories have been proposed to elucidate the development of multicystic ovaries in hypothyroid patients. One theory posits that the structural similarity between TSH and follicle-stimulating hormone (FSH) leads to ovarian FSH receptor stimulation by excessively elevated serum TSH levels in untreated hypothyroidism [65]. Another theory suggests that multicystic ovaries may arise from a disproportionate increase in FSH levels coupled with decreased luteinizing hormone (LH) concentrations, attributed to alterations in gonadotropin-releasing hormone (GnRH) pulse frequency mediated by elevated TRH levels in hypothyroid patients [66]. Additionally, hypothyroidism may entail reduced clearance of FSH, and there could be activating mutations in FSH receptors that augment the impact of elevated TSH levels on ovarian function [67].

Psychosis is documented in 5–15% of individuals with hypothyroidism and was initially documented as far back as 1888 by the Committee of the Clinical Society of London [68]. While the precise mechanism remains incompletely understood, both T4 and T3 play crucial roles in preserving proper neuronal conduction and cerebral blood flow [69,70].

Pseudoacromegaly presents as a clinical condition marked by physical features resembling those of growth hormone excess, yet with normal functioning of the somatotropic axis. Hypothyroidism induces the deposition and accumulation of hygroscopic mucopolysaccharides in the dermis and other tissues, occasionally resulting in acromegalic changes in the extremities and facial features. Although instances of pseudoacromegaly stemming from prolonged untreated primary hypothyroidism are infrequent in medical literature [20,37,71], the situation may become further complicated if pituitary enlargement is diagnosed, potentially leading to significant diagnostic and therapeutic challenges.

In our review, however, it cannot be excluded that the frequency of these more particular and rare symptoms is overestimated due to publication bias.

Although often considered asymptomatic, pituitary hyperplasia due to PH sometimes can be associated with some degree of hypopituitarism in almost 24% of patients in our review, similar to what was reported by Beck-Peccoz in 1996 [3]; in addition, visual field impairment can be present in more than one out of three patients.

The prevalence of hyperprolactinemia in PH has been reported to range from 0 to 42% in overt and from 8 to 20% in subclinical hypothyroidism [72,73,74,75,76,77]. In patients with PH, the degree of hyperprolactinemia tends to correlate with the severity of hypothyroidism [46,78], and this was also the case in our literature review, where TSH × ULN levels were higher in hyperprolactinemic patients. Generally, hyperprolactinemia is quite modest, with prolactin levels rarely exceeding 100 µg/L [46,76,77]. However, in our review, PRL levels > 100 µg/L were reported in 13.2% of patients, and in two patients, PRL levels exceeded 200 µg/L [33,35]. The pathophysiological mechanism underlying hyperprolactinemia in PH is primarily attributed to elevated TRH levels. In hypothyroidism, decreased thyroid hormone levels prompt increased TRH production by the hypothalamus. Elevated TRH levels can induce hyperplasia of both thyrotrophs and lactotrophs, leading to heightened PRL levels and an enhanced PRL response following intravenous TRH infusion [46,73,76,79]. Additional factors contributing to hyperprolactinemia in hypothyroid patients include reduced PRL clearance [80,81], diminished sensitivity to dopamine and dopamine agonists’ inhibitory effects [75,82], and potential elevation of macroprolactin [35]. Long-standing PH patients may develop pituitary hyperplasia, which could further exacerbate hyperprolactinemia by impeding hypothalamic dopamine’s ability to inhibit PRL secretion, known as the ‘stalk effect’. The presence of both hyperprolactinemia and pituitary gland enlargement may raise suspicion for a PRL-secreting pituitary adenoma, a non-functioning pituitary adenoma, or other sellar masses causing the ‘stalk effect’ and secondary PRL elevation.

During imaging, pituitary hyperplasia can be mistaken for a pituitary adenoma; this was the case in approximately 60% of our cases. Distinguishing between pituitary hyperplasia and pituitary adenoma is crucial, as their management is completely different; the former often responds to thyroid hormone replacement therapy, while the latter might need treatment with surgery and/or radiotherapy. However, even if pituitary hyperplasia is suspected and thyroid hormone replacement therapy is initiated, it is essential to closely monitor these patients with MRI scans to prevent overlooking a lesion that persists in growing despite hormone supplementation [60]. Such entities may be indistinct on imaging, but a mass induced by hyperplasia usually exhibits homogeneous enhancement and is indistinguishable from the normal gland [61]. Pituitary hyperplasia typically displays a homogeneous, symmetrical ‘dome-shaped’ architecture, unlike pituitary adenomas, which may vary in shape and homogeneity [9]. Analysis of pituitary images from our review also showed this typical ‘dome-shaped’ pituitary enlargement in 66% of cases. Ben-Shlomo et al. [83] have illustrated the anatomical location of thyrotrophs in the midline of the pituitary; therefore, any hyperplasia in the aforementioned region in patients suffering from PH could cause a typical ‘dome-shaped’ imaging characteristic. Thus, the presence of a ‘dome sign’ on pituitary MRI associated with an overt PH hormonal profile should suggest the presence of pituitary hyperplasia due to primary thyroid insufficiency rather than the presence of a pituitary adenoma. Consequently, these patients should be medically treated with levothyroxine, with the expectation of complete regression of the pituitary hyperplasia. The finding is also confirmed by our review, where the ‘nipple sign’ was present in 66% of cases. In the review from Beck-Peccoz and colleagues [3], there was no data about the role and frequency of the ‘nipple sign’ in pituitary hyperplasia; however, it is worth noting that the cases reported in our study were all studied using pituitary MRI and/or CT scans, compared to the 1996 review [3] where only two-thirds of the patients had baseline investigations represented by pituitary MRI or CT scans, and the percentage decreased to approximately 50% upon post-treatment reassessment. However, it is important to emphasize that also in the papers reviewed by us, the ‘nipple sign’ was explicitly documented in the text for only four patients, while in the remaining cases the data were inferred from direct imaging review. This underscores the limited attention still given today to this pathognomonic radiological sign of pituitary hyperplasia.

Yamada et al. [7] noted that the extent of enlargement of the sella turcica was associated with the rise in serum TSH levels. In one study, individuals with a TSH concentration exceeding 100 μIU/mL exhibited nearly a tenfold higher likelihood of having an enlarged pituitary compared to those with TSH levels ranging from 50 to 99 μIU/mL [34], suggesting a potential correlation between TSH levels and the presence of pituitary enlargement. Although we were unable to demonstrate a significant direct correlation between TSH levels (expressed as TSH × ULN) and pituitary dimensions (expressed as both maximal diameter and pituitary volume), it is noteworthy that pituitary volume was significantly higher in patients with TSH levels > 100 μIU/mL and in patients with a longer-standing diagnosis of hypothyroidism. Additionally, pituitary volume was found to be significantly greater in hyperprolactinemic patients compared to normo-prolactinemic ones, not only at diagnosis but also at follow-up.

These observations suggest that we have to pay close attention to patients with more severe decompensated primary hypothyroidism (TSH ≥ 100 μIU/mL), especially if long-standing and if associated with hyperprolactinemia. However, to date, it is not known whether a milder increase in TSH, as seen in subclinical hypothyroidism, which persists for a long time, can complicate with pituitary hyperplasia.

The main approach to managing pituitary hyperplasia secondary to PH involves administering thyroid hormone replacement therapy. It is crucial to closely monitor these patients for several reasons. Firstly, to confirm the diagnosis of pituitary hyperplasia due to PH, a reduction in pituitary size should be observed after initiating levothyroxine therapy. In general, when these patients are followed up, our review showed that a vast proportion (71.4%) of patients have a complete pituitary hyperplasia resolution, while in 22.1% at least a decrease in the size of the pituitary has been reported. These percentages are similar to those already reported by Beck-Peccoz in 1996 [3]. The timing of this improvement does vary in the literature, generally occurring even within one week in the case of an acute rise in thyroid hormone levels, but usually in several months [3]. Another reason for follow-up imaging is that, if there is no regression in the size of the pituitary despite adequate hormonal replacement therapy, the clinician should reconsider this diagnosis as a pituitary adenoma may be present. Consideration should be given to the possibility of a PRL-secreting adenoma if the size of the pituitary mass does not decrease during thyroxine therapy and if hyperprolactinemia persists, despite normalization of thyroid function. Moreover, the possibility of coexistence of overt PH with a non-functioning pituitary adenoma must also be considered; in this scenario, the lack of pituitary morphological response during levothyroxine therapy, despite normalization of the thyroid hormone profile, should raise diagnostic doubt. Even a TSH-secreting adenoma should not be ruled out, as this may be associated with PH: a TSH-secreting adenoma should be suspected if pituitary mass and TSH levels do not improve with thyroxine treatment. Thus, follow-up imaging and thyroid biochemistry are crucial for distinguishing between these conditions.

In our review, only one patient was deemed potentially affected by a non-functioning pituitary adenoma within the context of concurrent pituitary hyperplasia [40]. In a case of ongoing morphological stability and persistent hyperprolactinemia during therapy with L-thyroxine, suspicion of prolactinoma led to the subsequent initiation of bromocriptine therapy, with evidence of a subsequent response confirming the diagnostic hypothesis [46]. Although rare, a TSH-secreting adenoma was reported in a single case of concurrent pituitary hyperplasia [25]; in the other cases, the presence of a TSH-secreting pituitary adenoma (either pure or plurihormonal) appeared to be the only cause of the pituitary enlargement shown on MRI [22,39,43,51]. It remains to be clarified whether such adenomas could arise from the progression of a pituitary hyperplasia sustained by a long-standing PH, followed by pituitary TSH-secreting cell autonomization.

Considering the role of neurosurgery in pituitary hyperplasia due to PH, it is generally reserved for patients with visual field defects who require optic chiasm decompression to prevent permanent vision loss. Additionally, if the pituitary gland continues to enlarge despite levothyroxine treatment, neurosurgery may be necessary to confirm the diagnosis through pathological examination [84]. However, based on our review data, very few patients required surgery due to compressive symptoms or an inadequate response to levothyroxine replacement therapy.

## 5. Conclusions

The association between pituitary hyperplasia and PH should be kept in mind when pituitary enlargement is detected on MRI before unwarranted and drastic interventions are initiated.

In the context of untreated PH, pituitary hyperplasia, which is reversible with thyroid hormone replacement therapy, is the primary etiological diagnosis of a pituitary mass. However, occasionally a TSH-secreting pituitary tumor may develop within a hyperplastic gland, though its autonomy remains unclear. Additionally, an incidental pituitary tumor unrelated to PH is also possible. These alternative diagnoses should be considered if the pituitary mass does not adequately resolve with appropriate thyroid hormone replacement.

A multidisciplinary team approach (a primary clinician, an endocrinologist, a neurosurgeon, and an ophthalmologist) is preferred in the management of patients with pituitary hyperplasia secondary to overt PH. The goal of the team is to provide the patient with optimal care in the timeliest fashion overall.

## Figures and Tables

**Table 1:** Thyroid function profile trend during the administration of 1000 µg of levothyroxine in a weekly oral single dose under medical supervision.

	TSH (0.35–4.94 µIU/mL)	fT4 (7–14.8 ng/L)	fT3 (1.6–3.9 ng/L)
Baseline	47	5	2
1st week	23.1	8	2.4
2nd week	5.41	8	2.6
3rd week	2.73	8	2.9
4th week	0.94	9	2.5

**Table 2:** Symptoms that led to the diagnosis of pituitary hyperplasia in 58 out of the 93 analyzed patients in whom symptoms were reported at diagnosis.

Symptom	*n*			%		
	All (*n* = 58)	M (*n* = 12)	F (*n* = 46)	All (100%)	M (20.7%)	F (79.3%)
Headache and/or visual disturbance	28	7	21	48.3	58.3	45.7
Hypothyroidism	27	5	22	46.6	41.7	47.8
Irregular menses	23	NA	23	NA	NA	50.0
Galactorrhea	13	NA	13	NA	NA	28.3
Primary ovarian hyperstimulation syndrome	6	NA	6	NA	NA	13.0
Psychosis	2	0	2	3.4	0	4.3
Pseudoacromegaly	2	2	0	3.4	16.7	0
Cretinism	1	1	0	1.7	8.3	0
Others *	6	1	5	10.3	8.3	10.9

NA: not applicable; M: male; F: female. * One case with sudden attack of lancinating pain into the right jaw and neck, right hemiparesis, and hemisensory loss that lasted for one hour and resolved spontaneously (F); one case with abdominal pain and diarrhea (F); one case of loss of consciousness and epileptic seizure (F); one evaluation performed for dubious inappropriate TSH secretion (F); one case of palsy of the seventh cranial nerve (F); one case with right nasal obstruction and two attacks of epistaxis (M).

**Table 3:** CT and MRI imaging at diagnosis in 93 patients with pituitary hyperplasia due to longstanding primary hypothyroidism.

CT and MRI Imaging at Diagnosis	Yes (*n*)	No (*n*)	Not Specified (*n*)
Mass with only intrasellar extension	3	57	33
Mass with suprasellar extension	56	2	35
Mass with invasion of the cavernous sinus	10	48	35
Mass with infrasellar extension	6	52	35
Optic chiasm compression	38	16	39
Dome-shaped architecture	24	12	57

**Table 4:** Type of treatment for pituitary mass in 93 patients with pituitary hyperplasia due to longstanding primary hypothyroidism.

Type of Treatment	*n*	%
Levothyroxine	76	81.7
Neurosurgery	1	1.1
Triiodothyronine	1	1.1
Levothyroxine + Neurosurgery	10	10.7
Not specified	5	5.4

## Data Availability

The protocol and the raw data supporting the conclusions of this article will be made available by the authors upon request.

## Supplementary Materials

The following supporting information can be downloaded at: https://www.mdpi.com/article/10.3390/biomedicines12061368/s1. Table S1: Table summarizing the characteristics and main data of the analyzed studies.
