# Supplementary material for: Pituitary Hyperplasia Due to Longstanding Primary Hypothyroidism: A Case Report and Comprehensive Review of the Literature
Source: Biomedicines. 2024 Jun 19;12(6):1368. doi: 10.3390/biomedicines12061368 (PMC11202140; doi:10.3390/biomedicines12061368)
Supplement: Supplementary file 1 [file biomedicines-12-01368-s001.zip › biomedicines-3025032-supplementary.pdf]

Table S1: Table summarizing the characteristics and main data of the analyzed studies

First Author, Year of Publication, [Reference No.]	Gender	Age (yrs)	TSH (µIU/mL)	TSH normal value (µIU/mL)	PRL (µg/L)	PRL normal value (µg/L)	Baseline pituitary dimension	Treatment	MRI outcome
Agerd L, 2014, [12]	F	41	>100	0.3-3.0	30.21	NA	NA	levothyroxine	CR
Alkhani A.M, 1999, [13]	F	51	>100	0.4-5.5	90	0-25.0	16x12x10 mm 960 mm^3	levothyroxine + NS	NA
Ansari M.S., 2016, [14]	F	67	863.3	0.2-4.2	154	4.6-23.6	19x16x18 mm 2736 mm^3	levothyroxine	PR
Ban Y., 2000, [15]	F	40	293	0.35-3.73	56	<15	15 mm	levothyroxine	CR
	F	46	33.8	0.35-3.73	NA	NA	NA	NA	NA
	F	74	202	0.35-3.73	11.3	<15	NA	NA	NA
	F	46	1600	0.35-3.73	27.6	<15	NA	NA	NA
	M	44	235	0.35-3.73	7.4	<15	NA	NA	NA
Bassam T., 2006, [16]	F	19	4191.5	0.47-5.01	38.1	3.8-23.2	11 mm 1567 mm^3	levothyroxine	CR
Bhansali A., 2004, [17]	F	19	120	0.5-6.5	58.5	5.0-25.0	14x10x12 mm 840 mm^3	levothyroxine	CR
Bhansali A., 2004, [18]	F	19	357.7	0.5-5.4	128	5.0-25.0	40x43x37 mm 31820 mm^3	levothyroxine	CR
Brändle M., 2000, [19]	F	21	506.75	0.1-4.0	118	2.0-20.0	12x10 mm	levothyroxine	CR
Chakraborty P.P, 2017, [20]	M	64	>75	NA	33.4	NA	NA	NA	NA
Chakraborty S., 2020, [9]	F	24	>100	NA	NA	NA	13x10x21 mm 1365 mm^3	NS	NA
De Sousa, S.M., 2015, [21]	F	19	566	0.4-3.5	37.2	4.0-23.8	13 mm	levothyroxine	PR
Du J., 2020, [22]	M	35	100	0.27-4.2	38.1	2.6-13.2	18x14x13 mm 1638 mm^3	levothyroxine + NS	NA
	F	56	100	0.27-4.2	34.4	2.6-13.2	NA	levothyroxine + NS	NA
Eiland L., 2012, [23]	F	23	1398	0.34-5.6	60	2.1-49.7	10x21 mm	levothyroxine	CR
Erem C., 2010, [24]	F	21	100	0.27-4.2	97	3.4-24.1	18x16x14 mm 2016 mm^3	levothyroxine	CR
Ghannam N.N., 1999, [25]	M	41	3474	0.35-5.5	<2.0	2.1-17.7	NA	levothyroxine + NS	PR
Goswami R., 1999, [26]	M	25	489	0.3-4.0	18	NA	14 mm	levothyroxine	PR
Gutch M., 2017, [27]	F	28	>300	0.3-5.5	3.1	NA	NA	levothyroxine	NA
Haider S., 2023, [28]	F	29	1600	0.47-4.2	114	4.8-23.3	23x18x12 mm 2484 mm^3	levothyroxine	CR
Hoogenberg K., 2003, [29]	M	51	120	NA	27.6	2.4-9.5	12 mm	levothyroxine	CR
Jentoft M.E., 2012, [6]	F	40	800	0.4-4.5	54	3.0-30.0	10 mm	levothyroxine + NS	NA
Johnston P.C., 2014, [30]	F	20	1160.9	0.3-4.2	116.2	<21.4	15.4 mm	levothyroxine	CR
Joshi A.S., 2005, [31]	F	29	177	NA	14	NA	12 mm	triiodothyronine	S
Kanza R.E., 2013, [32]	F	19	100	0.47-4.68	42	3.0-18.0	13x8x10 mm 520 mm^3	levothyroxine	CR
Katulande P., 2013, [33]	F	23	>100	0.5-5.0	4095	<20	15x10x12 mm 900 mm^3	levothyroxine	NA
Khawaja N.M., 2006, [34]	F	19	65	0.47-5.01	16	3.8-23.2	12 mm 1080 mm^3	levothyroxine	PR
	F	46	69	0.47-5.01	NA	3.8-23.2	14 mm 539 mm^3	levothyroxine	CR
	F	41	71	0.47-5.01	24	3.8-23.2	12 mm 672 mm^3	levothyroxine	CR
	F	32	>100	0.47-5.01	49	3.8-23.2	11 mm 1188 mm^3	levothyroxine	CR
	F	33	>100	0.47-5.01	16	3.8-23.2	10 mm	levothyroxine	CR

							420 mm^3		
	F	40	>100	0.47-5.01	11	3.8-23.2	12 mm<br>600 mm^3	levothyroxine	CR
	F	32	>100	0.47-5.01	69	3.8-23.2	11 mm<br>726 mm^3	levothyroxine	CR
	F	32	>100	0.47-5.01	33	3.8-23.2	11 mm<br>1045 mm^3	levothyroxine	PR
	F	28	>100	0.47-5.01	12	3.8-23.2	12 mm<br>1482 mm^3	levothyroxine	PR
	F	30	120	0.47-5.01	NA	3.8-23.2	20 mm<br>1000 mm^3	levothyroxine	CR
	F	19	145	0.47-5.01	16	3.8-23.2	10 mm<br>720 mm^3	levothyroxine	S
	F	30	157	0.47-5.01	50	3.8-23.2	15 mm<br>1072 mm^3	levothyroxine	S
	M	56	190	0.47-5.01	12	3.8-23.2	10 mm<br>599 mm^3	levothyroxine	CR
	F	32	191	0.47-5.01	NA	3.8-23.2	12 mm<br>1404 mm^3	levothyroxine	CR
	F	26	195	0.47-5.01	36	3.8-23.2	13 mm<br>585 mm^3	levothyroxine	I
	F	20	230	0.47-5.01	NA	3.8-23.2	13 mm<br>1040 mm^3	levothyroxine	CR
	F	33	261	0.47-5.01	87	3.8-23.2	11 mm<br>1100 mm^3	levothyroxine	PR
	F	65	269	0.47-5.01	28	3.8-23.2	13 mm<br>312 mm^3	levothyroxine	CR
	F	26	632	0.47-5.01	34	3.8-23.2	13 mm<br>1040 mm^3	levothyroxine	PR
Khorassanizadeh R., 2016, [35]	F	30	263	0.36-3.74	323	2.2-30.3	904.8 mm^3	levothyroxine	NA
Kroese J.M., 2004, [36]	F	36	>75	0.4-4.0	103	0-20.0	14x20x12 mm<br>1680 mm^3	levothyroxine	CR
Kumar K.V., 2012, [37]	M	22	122.2	0.3-4.5	24	0-15.0	16x11 mm	levothyroxine	CR
Lipkes C., 2022, [38]	F	29	>1000	NA	NA	NA	23 mm	levothyroxine	CR
Ma W., 2003, [39]	F	26	1329.4	NA	31.1	NA	NA	levothyroxine + NS	CR
Mahesh D.M., 2015, [40]	M	43	775	0.5-4.5	NA	NA	71x59x42 mm<br>87969 mm^3	levothyroxine	PR
Martinez Quintero B., 2020, [41]	F	53	247	NA	NA	NA	11x8x8 mm<br>352 mm^3	levothyroxine	CR
Moumen A., 2015, [42]	M	35	>100	0.34-5.6	22.5	2.64-13.13	15x17x12 mm<br>1530 mm^3	levothyroxine	CR
Myers A., 2011, [43]	F	54	6.5	0.4-4.5	38	4.8-23.3	23x24x24 mm<br>6624 mm^3	levothyroxine + NS	NA
Nachawi N., 2018, [44]	F	in her 30s	185	NA	normal value	NA	9x10x13 mm<br>585 mm^3	levothyroxine + NS	NA
Neves C.P., 2015, [45]	F	21	>500	0.4-4.3	47.6	0-33.3	19x17x12 mm<br>1938 mm^3	levothyroxine	PR
Notsu K., 1997, [46]	F	69	7.1	0.3-4.7	123	2.3-13.4	NA	levothyroxine	S
	F	26	1198	0.3-4.7	91	2.3-13.4	NA	levothyroxine	CR
Ozbey N., 1997, [47]	F	32	97	0.4-4.0	77	2.0-20.0	17x15 mm	levothyroxine	CR
Pappy A.L., 2016, [48]	F	63	150.8	0.4-4.5	19.2	NA	NA	levothyroxine	CR
Passeri E., 2011, [49]	F	23	1578	NA	113	NA	23x23x10 mm<br>2645 mm^3	levothyroxine	CR
Quintyne K.I., 2010, [50]	M	33	85.1	0.15-3.2	10	1.4-23.8	14x10x12 mm<br>840 mm^3	levothyroxine	CR
Ren X., 2023, [51]	M	49	150	0.34-5.5	13.65	2.64-13.3	NA	levothyroxine + NS	CR
Sansone A., 2017, [52]	F	29	665.5	0.5-4.0	30	5.0-27.0	11x8x7 mm<br>308 mm^3	levothyroxine	CR
Sarlis N.J., 1997, [53]	F	26	563.3	0.4-4.4	95.4	1.0-24.0	12 mm	levothyroxine	CR

Sharma A., 2020, [54]	F	32	512	0.35-4.0	NA	NA	18x15 mm	levothyroxine	PR
Shimono T., 1999, [55]	F	62	31.6	0.3-3.9	8.9	1.4-14.6	396 mm^3	levothyroxine	CR
	M	68	49.4	0.3-3.9	6.7	1.5-9.7	539 mm^3	levothyroxine	CR
	F	69	115.5	0.3-3.9	6.9	1.4-14.6	375 mm^3	levothyroxine	CR
	F	62	78.8	0.3-3.9	8.4	1.4-14.6	308 mm^3	levothyroxine	CR
	F	63	47	0.3-3.9	6	1.4-14.6	338 mm^3	levothyroxine	CR
	F	50	39.5	0.3-3.9	7.2	1.4-14.6	539 mm^3	levothyroxine	CR
	M	67	69	0.3-3.9	6	1.5-9.7	539 mm^3	levothyroxine	CR
	F	46	84	0.3-3.9	29.1	1.4-14.6	501 mm^3	levothyroxine	CR
	M	46	115	0.3-3.9	12.4	1.5-9.7	561 mm^3	levothyroxine	CR
	M	66	53	0.3-3.9	7.6	1.5-9.7	546 mm^3	levothyroxine	CR
	M	72	61	0.3-3.9	12.3	1.5-9.7	312 mm^3	levothyroxine	CR
	M	32	186.5	0.3-3.9	8.9	1.5-9.7	600 mm^3	levothyroxine	CR
	F	79	82.5	0.3-3.9	8.6	1.4-14.6	300 mm^3	levothyroxine	CR
	F	83	106.5	0.3-3.9	10.9	1.4-14.6	490 mm^3	levothyroxine	CR
Shukla P., 2019, [56]	F	34	251.21	0.5-5.5	29.48	3.34-26.72	14x12x13 mm<br>1092 mm^3	levothyroxine	PR
Siddiqi A.I., 2015, [57]	M	18	>100	0.27-4.2	54	4.1-15.4	NA	levothyroxine	PR
Shekhawat V.S., 2021, [58]	M	34	731.8	0.3-5.5	64.2	2.0-20.0	NA	levothyroxine	CR
Taher B.M., 2004, [59]	F	22	>100	0.47-5.01	71.3	3.8-23.2	8.6x11.5x20 mm<br>989 mm^3	levothyroxine	NA
Vecil G.G., 2008, [60]	F	26	210	0.5-5.5	34.9	2.8-29.2	8x8 mm	levothyroxine	CR
Wolansky L.J., 1996, [61]	F	27	283	0-3.0	53	0-20.0	17 mm	levothyroxine	PR
Yamashita Y., 2001, [62]	F	19	132.75	0.48-4.82	61.5	0-26.3	NA	levothyroxine	CR
Young M., 1999, [63]	F	25	88.1	0.4-6.0	48.3	3.0-24.0	NA	levothyroxine + NS	CR
Zhang H., 2014, [64]	F	19	100.85	NA	137.91	NA	30x30x30 mm<br>13500 mm^3	levothyroxine	PR

F: female; M: male; NA: not available; NS: neurosurgery; CR: complete resolution; PR: partial resolution; S: stability; I: increase
